# Lipoprotein turnover and possible remnant accumulation in preeclampsia: insights from the Freiburg Preeclampsia H.E.L.P.-apheresis study

**DOI:** 10.1186/s12944-018-0698-4

**Published:** 2018-03-14

**Authors:** Christine Contini, Martin Jansen, Brigitte König, Filiz Markfeld-Erol, Mirjam Kunze, Stefan Zschiedrich, Ulrich Massing, Irmgard Merfort, Heinrich Prömpeler, Ulrich Pecks, Karl Winkler, Gerhard Pütz

**Affiliations:** 1 Institute of Clinical Chemistry and Laboratory Medicine, Medical Center - University of Freiburg, Hugstetter Straße 55, 79106 Freiburg, Germany; 2 Department of Obstetrics and Gynecology, Medical Center - University of Freiburg, Hugstetter Straße 55, 79106 Freiburg, Germany; 3 Department of Medicine IV (Nephrology and Primary Care), Medical Center, University of Freiburg, Hugstetter Straße 55, 79106 Freiburg, Germany; 4 Andreas Hettich GmbH & Co KG, Engesser Straße 4a, 79108 Freiburg, Germany; 5 Department of Pharmaceutical Biology and Biotechnology, Albert-Ludwigs-University of Freiburg, Stefan-Meier-Straße 19, 79104 Freiburg, Germany; 6 Department of Gynecology and Obstetrics, University Hospital Schleswig-Holstein Campus, Kiel, Germany

**Keywords:** Preeclampsia, Hypertension in pregnancy, Apheresis, Lipoprotein removal, Remnant lipoproteins

## Abstract

**Background:**

Preeclampsia is a life-threatening disease in pregnancy, and its complex pathomechanisms are poorly understood. In preeclampsia, lipid metabolism is substantially altered. In late onset preeclampsia, remnant removal disease like lipoprotein profiles have been observed. Lipid apheresis is currently being explored as a possible therapeutic approach to prolong preeclamptic pregnancies. Here, apheresis-induced changes in serum lipid parameters are analyzed in detail and their implications for preeclamptic lipid metabolism are discussed.

**Methods:**

In the Freiburg H.E.L.P.-Apheresis Study, 6 early onset preeclamptic patients underwent repeated apheresis treatments. Serum lipids pre- and post-apheresis and during lipid rebound were analyzed in depth via ultracentrifugation to yield lipoprotein subclasses.

**Results:**

The net elimination of Apolipoprotein B and plasma lipids was lower than theoretically expected. Lipids returned to previous pre-apheresis levels before the next apheresis even though apheresis was repeated within 2.9 ± 1.2 days. Apparent fractional catabolic rates and synthetic rates were substantially elevated, with fractional catabolic rates for Apolipoprotein B / LDL-cholesterol being 0.7 ± 0.3 / 0.4 ± 0.2 [day^− 1^] and synthetic rates being 26 ± 8 / 17 ± 8 [mg*kg^− 1^*day^− 1^]. The distribution of LDL-subclasses after apheresis shifted to larger buoyant LDL, while intermediate-density lipoprotein-levels remained unaffected, supporting the notion of an underlying remnant removal disorder in preeclampsia.

**Conclusion:**

Lipid metabolism seems to be highly accelerated in preeclampsia, likely outbalancing remnant removal mechanisms. Since cholesterol-rich lipoprotein remnants are able to accumulate in the vessel wall, remnant lipoproteins may contribute to the severe endothelial dysfunction observed in preeclampsia.

**Trial registration:**

ClinicalTrails.gov, NCT01967355.

## Background

Preeclampsia (PE) is usually defined as the new onset of hypertension (> 140/90 mm/Hg) and proteinuria (> 300 mg/24 h) occurring after the 20th week of pregnancy [1]. Unfortunately, PE’s pathophysiology remains elusive, and it is still a life-threatening disease for mother and fetus [2]. Women with PE are at risk of inflammation and endothelial damage in multiple organ systems [3] while the fetus is at risk of intrauterine growth restriction and/or placental abruption [4]. Preeclampsia is associated with a two-fold increased risk of perinatal death [5]. PE is probably a multicausative disease characterized by placental malfunction (but not necessarily leading to intrauterine growth restriction) and several pathological imbalances [6, 7]. In response to fetal demands, triglycerides and cholesterol rise in all lipoprotein fractions during normal pregnancy [8–10]. Even more pronounced changes in lipoprotein metabolism are observed in PE [11, 12]. Preeclamptic pregnancies show higher levels of triglyceride-rich lipoproteins [13] and the highest triglyceride levels in pregnancy correlate with a four-fold increased risk for PE [14].

The only causative therapy for PE today consists in delivery [4] with all the risks of a premature delivery for the fetus – especially in early-onset PE before the 34th week of gestation. In early-onset PE, any treatment that prolongs pregnancy would substantially enable the fetus to mature further and is considered beneficial. Despite intensive research over the last decades, established therapeutic options for PE are still lacking, not least because of the immanent difficulties in performing clinical studies in pregnant women. Nevertheless, several successful studies have recently drawn attention to lipid-apheresis as a possible therapeutic approach in preeclampsia [15–17]. Based on the rationale that high lipid levels can trigger endothelial dysfunction and that lipid-apheresis improves endothelial function, Wang et al. [17] published the first pilot study using heparin-mediated extracorporeal LDL-precipitation (H.E.L.P.-apheresis) in nine pregnant women with PE. Dextrane-sulfate cellulose apheresis (DSC-apheresis) was successfully used in two studies with 14 preeclamptic patients altogether [15, 16]. The present work is based on a recent study using H.E.L.P.-apheresis with six patients [18]. Both apheresis techniques used so far are primarily designed to reduce Apolipoprotein B (ApoB) containing plasma lipoproteins such as very-low-density lipoprotein (VLDL) and low-density lipoprotein (LDL) by 50–60% [19–22]. Both techniques are not specific and remove a wide variety of pro-inflammatory and pro-coagulatory substances as well, and a possible role of the additional elimination of sFlt by DSC is currently controversially discussed [18, 23].

To study lipoprotein dynamics, stable isotope kinetic procedures are considered the gold standard, but they are unfortunately unfeasible in pregnant women. Apheresis substantially alters lipid levels and offers the unique opportunity to investigate lipid-metabolism dynamics without stable isotope kinetic procedures [24]. A deeper understanding of pathophysiological alterations in lipid metabolism during PE may allow better understanding of PE pathophysiology ultimately leading to better therapeutic care. In the present work, we analyzed alterations in the various lipoprotein classes in pregnant women with PE undergoing apheresis in the “Freiburg Preeclampsia H.E.L.P. Apheresis study” [18] in detail, and discuss possible implications for altered lipid metabolism during PE.

## Methods

### Participants and study protocol

Pregnant women with early-onset preeclampsia (≤ 32 gestational weeks) fulfilling the criteria of the German Society for Obstetrics and Gynecology (August 2010): hypertension and proteinuria (≥ 1 + dipstick or ≥300 mg/24 h) with or without intrauterine growth retardation were eligible for the study. Other inclusion criteria were age over 18 years, pathological Doppler evaluation and informed written consent. During the study, all clinical procedures and therapies were continued as clinically indicated, and pregnancies could be terminated at any time if necessary. Apheresis-treatments were planned individually according to the clinical situation.

We had access to a total of 23 full datasets of lipid profile analysis obtained from six pregnant women with PE who underwent 2–6 H.E.L.P.-apheresis at the Medical Center - University of Freiburg. As this was a pilot study, patients were not randomized to a treatment or control group.

The study was approved by the local ethics committee at the Medical Center - University of Freiburg, (local ethics committee number: 475/12; ClinicalTrails.gov: NCT01967355), and all patients gave informed written consent.

### H.E.L.P.-apheresis

H.E.L.P.-apheresis was performed with the H.E.L.P. Plasmat Futura© (BBraun Melsungen) according to standard operating protocols. After a bolus injection of unfractionated heparin, plasma was separated from blood cells and acetate buffer and heparin were added. ApoB-containing lipoproteins precipitated with heparin at acidic pH and were filtered through a polycarbonate filter. The filtrate was then dialyzed against a bicarbonate solution and returned together with the blood cells into the patient [25]. Plasma-EDTA samples were taken immediately before and after the apheresis treatments.

### Sequential ultracentrifugation

Plasma samples were subjected to preparative sequential density ultracentrifugation with a target density less than 1.006 kg/l for very low-density lipoprotein (VLDL), between 1006 and 1.019 kg/l for IDL, between 1019 and 1.063 kg/l for LDL, between 1063 and 1.21 kg/l for HDL as previously described [13]. LDL subfractions were separated according to Baumstark et al. [26]. LDLs were separated further into six classes with different densities: LDL-1, 1.019–1.031 kg/l; LDL-2, 1.031–1.034 kg/l; LDL-3, 1.034–1.037 kg/l; LDL-4, 1.037–1.040 kg/l; LDL-5, 1.040–1.044 kg/l; LDL-6, 1.044–1.063 kg/l.

### Determination of lipid parameters

ApoB, ApoA1, cholesterol and triglycerides were measured on an autoanalyzer platform (Olympus AU 640) with the respective commercially available test kits according to the manufacturer’s instructions (DiaSys Greiner). Lipid concentrations are given in mg/dL.

### Calculations

#### Plasma volume

Plasma volume before the first apheresis was estimated from each patient’s weight, height, and hematocrit [27] (http://lipid-apherese.info/plasmarechner.htm; accessed 05.08.2016).

#### Apheresis efficiency

Calculated reduction rates depend on the plasma volume treated and patient’s total plasma volume. To estimate the reduction during apheresis treatment, we applied Eq. 1 [25]:

1 $$ \frac{conc_{after\ apheresis}}{conc_{before\ apheresis}}=\mathit{\exp}\left(-K\frac{plasmavolume_{treated}}{plasmavolume_{total}}\right) $$

In Eq. 1, conc gives the concentration of the respective analyte and the factor K describes the elimination efficiency for the respective species. Since the filtrate is essentially ApoB-free, the factor *K = 1* for ApoB and LDL-cholesterol while for HDL-cholesterol *K < 0.18* [25].

#### Fractional catabolic rate (FCR) and synthetic rate (SR)

Assuming a constant production rate and a first order elimination rate in steady state, Eq. 2 allows the calculation of an apparent FCR’ by rebound data [24]. FCR calculated by rebound data correlated decently with stable isotope-derived FCR in mean respective median, it did not necessarily correlate well on the individual level [28], thus the calculated FCR are referred to as apparent FCR’ in this work:

2 $$ \mathit{\ln}\left[\frac{Conc_0-{Conc}_t}{Conc_0-{Conc}_{min}}\right]=- kt $$

[24] where conc_´0_ is the concentration of the analyte before apheresis, conc_min_ is the concentration immediately after apheresis, conc_t_ is the measured concentration at time t; and k = FCR’.

Apparent synthetic rate SR’ was estimated as a product of FCR’ and plasma pool size expressed per kilogram of body weight [17] and calculated according to the following equation:

3 $$ {SR}^{\prime }={FCR}^{\prime}\frac{preapheresis\ level\left[ mg/ dL\right]\ast plasma\ volume\left[ dl\right]}{weight\left[ kg\right]} $$

To assess individual FCR’ for each patient, for each measured time point after first apheresis, Eq. 2 was used to calculate k, and FCR’ was given as mean of all measurements taken into account for this patient in Table 3. From FCR’, respective SR’ were calculated by Eq. 3. For Fig. 3, all apheresis treatments were taken into account, and the constant k was computed by fitting a first order exponential function to the measured rebound data. The rebound half time R_50_ was calculated according to ln(2)/k.

### Statistical analysis

Single datasets before and after apheresis were analyzed with Wilcoxon matched pairs test. LDL-subfractions were tested with multiple t-tests with Holm-Sidak post-test for unpaired analysis and with Wilcoxon matched pairs test for comparison of calculated values and measured values. A value of *p* < 0.05 was considered statistically significant. Statistical analyses were done using GraphPad Prism Software V7.00.

## Results

### Baseline characteristics and apheresis treatments

Table 1 illustrates the baseline characteristics of our six eligible patients. Mothers recovered from preeclamptic symptoms completely after delivery. All babies received intensive neonatal care and were discharged in healthy condition. A detailed discussion of the clinical outcomes is given in [18]. Despite the severe clinical conditions, apheresis was generally well-tolerated and we observed only minor complications throughout the 23 aphereses performed: On one occasion, apheresis treatment had to be terminated due to an extravasation. On two occasions, patients suffered from a blood pressure drop, which was treated by saline infusion. On one occasion, a clot formed in the tubing at the end of treatment.

**Table 1:** Characteristics of study patients

Patient	Age	Gestational age^a^at admission	RR^b^ at admission	Number of apheresis treatments	Gestational age^a^at delivery	Time to delivery (days)	Birth Weight	Baby survived/discharged in healthy condition?
1	41	24 + 4	170/100	3	27 + 5	22	860 g	Yes/Yes
2	35	24 + 4	157/89	5	26 + 6	16	660 g	Yes/Yes
3	31	25 + 2	152/97	4	27 + 4	16	430 g	Yes/Yes
4	31	25 + 3	148/97	6	28 + 0	18	835 g	Yes/Yes
5	30	26 + 4	142/92	3	28 + 0	10	530 g	Yes/Yes
6	32	27 + 0	147/88	2	28 + 1	8	520 g	Yes/Yes

^a^Gestational age in weeks+days

^b^Blood pressure after Riva Rocci in mmHg

### Lipid levels before and after apheresis

**Fig. 1:**
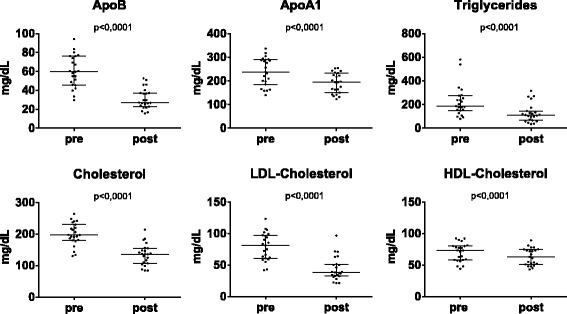
Serum lipid levels before and after apheresis. Samples were taken immediately before and after apheresis treatments (in total *n* = 23 treatments). Values before and after apheresis are given as median and interquartile range and tested with Wilcoxon matched pairs test

All lipid parameters were significantly reduced by apheresis (Fig. 1). As H.E.L.P.-apheresis addresses primarily ApoB-containing lipoproteins, HDL-cholesterol and ApoA1 were reduced to a much lower proportion than ApoB and ApoB-related lipids. Apheresis efficiency depends on the ratio of treated plasma volume to total plasma volume (see Eq. 1). In general, plasma volume is elevated during pregnancy [29], but reduced plasma volume is not uncommon in PE [30]. In the early-onset PE patients included in this study, plasma volume was rather similar to non-pregnant individuals (Table 2). On average we treated 0.9 plasma volumes. Table 2 compares the measured reduction rates with the average reduction rates of apheresis treatments derived from the literature and individually calculated reduction rates according to theoretically expected treated plasma volumes. Decrease in blood levels of ApoB, LDL-cholesterol and triglycerides were significantly lower than those calculated and lower than those observed in other pathologies treated by LDL-apheresis.

**Table 2:** Characteristics of H.E.L.P.-apheresis. The characteristics of H.E.L.P.-apheresis in patients treated in this study (*n* = 6) are compared to theory (calculated values) and literature

	Preeclampsia measured^a^ (Freiburg)	Preeclampsia measured Wang et al. [17]	Preeclampsia c*alculated*^*b*^	H.E.L.P. literature^a^
Plasma treated [ml]	2566 ± 361	–	2566	2500–3000 [19, 20]
ApoB reduction [%]	−47 ± 14%	–	− 59 ± 5	- 63% [31], − 54% [21]
LDL-cholesterol reduction [%]	−43 ± 17%	−44%	−59 ± 5	−67% [22], − 53% [21]
HDL-cholesterol reduction [%]	−7 ± 5%	−15%	− 15 ± 2	− 15% [22], − 12% [21]
Triglycerides reduction [%]	−42 ± 14%	−41%	–	−41% [22], − 55% [21], − 60% [19]
ApoA1 reduction [%]	−17 ± 5%	–	–	− 16% [21]
Total plasma volume [ml]	2827 ± 225^c^		2827^c^	2638 ± 414 (non-pregnant) [50] 3216 ± 168 (normotensive pregnancy) [29]
Plasmaflow [ml/min]	25 ± 3		–	20–30 [20]
Duration of apheresis [min]	123 ± 17		–	100–150 [20]

^a^As occurring or worsening edema during therapy interferes with calculating the plasma volume, only data from each patient’s first apheresis were considered; ^b^Theoretical reduction was calculated in consideration of the plasma volume and treated plasma volume according to Eq. 1

### Rebound of lipids after apheresis

**Fig. 2:**
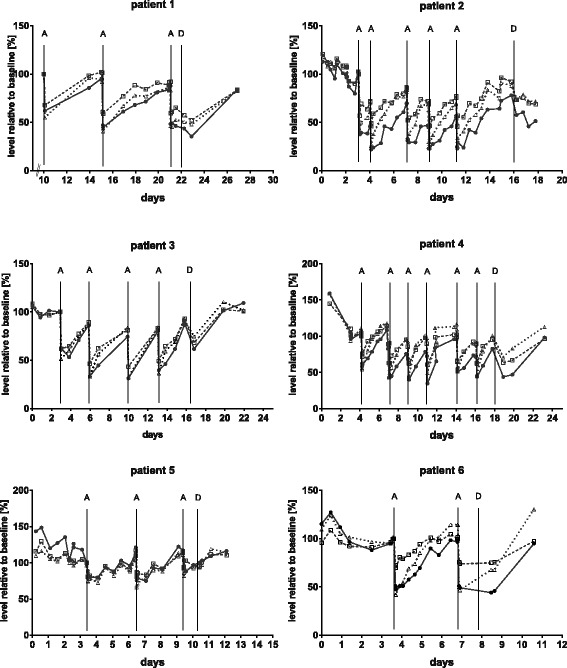
Rebound of lipid levels after apheresis treatments in the six PE patients. Lipid levels before the first apheresis were set as 100%, all other lipid values are given in % relative to this value. A: apheresis treatment, D: delivery. Closed circles: LDL-cholesterol; open rectangles: total cholesterol; open triangles: ApoB

**Fig. 3:**
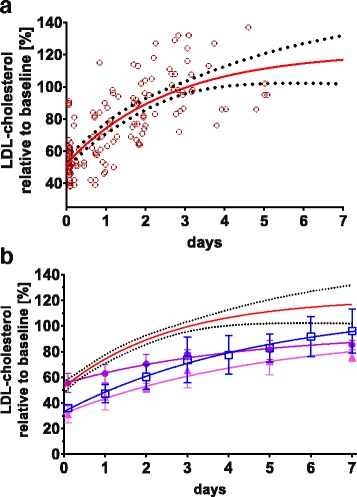
Rebound of LDL-cholesterol. **a**: LDL-cholesterol measurements after H.E.L.P.-apheresis for all apheresis treatments in this study until day 5 (red open circles). Values before apheresis were set to 100% and all other values are given in % relative to this value, starting with the first value immediately after apheresis. The red line depicts a monoexponential fit of all data (*r* = 0.55), the black dotted line gives the 95% CI of the computed graph. **b**: Rebound of LDL-cholesterol after H.E.L.P.-apheresis, literature data (mean and s.d.). Blue open squares: 6 otherwise healthy subjects presenting isolated high Lp(a) levels [31]; magenta circles 4 healthy subjects, 75% of plasma volume treated; magenta triangles, 4 healthy subjects, 125% plasma volume treated [49]

Apheresis treatments were performed at intervals between 2 to 4 days, depending on the clinical situation. Within this time frame, lipids regained previous pre-apheresis levels before the next apheresis (99 ± 16% for ApoB, 97 ± 25% for LDL-cholesterol and 100 ± 13% for total cholesterol after 2.9 ± 1.2 days). Figure 2 shows the individual rebound of different lipid parameters after apheresis; Fig. 3a depicts all the rebound data obtained in this study until day five. A monoexponential function was fitted to these data and the rebound half-time R_50_ calculated to 1.8 (95% CI 1.1 to 2.4) days. Since carrying out an intervention like apheresis in healthy pregnant individuals is out of question, we compared the observed rebound data to historical apheresis data. In six otherwise healthy patients with normal LDL cholesterol and isolated elevated Lp(a) [31], LDL-cholesterol reached pre-apheresis levels on average after seven to eight days (Fig. 3b). Respective R_50_ was calculated to 3.4 (95% CI 1.5 to 5.3) days. Although only 75% of plasma volume had been treated, normal healthy patients (*n* = 4) did not reach pre-apheresis levels within seven days [32], and respective R_50_ was calculated to 3.0 (95% CI from 2.2 to 12.5) days when a minimum of 95% of pre-apheresis level was assumed. Taken together, serum lipids in PE patients seem to rebound much faster than they do in individuals with normal ApoB metabolism.

Each patient’s apparent FCR’ were calculated applying Eq. 2 (Table 3). Only the first apheresis treatments were considered, since occurring or worsening edema during therapy may interfere with calculating the plasma volume. Interestingly, FCR calculated from k derived by first exponential fitting of all apheresis treatment data available for LDL-cholesterol (0.49 (95% CI 0.27 to 0.76) [day^− 1^] (Fig. 3), gave similar results as individual calculations of first apheresis treatments (0.43 ± 0.22 [day^− 1^] (Table 3), indicating no change in plasma volume and lipid metabolism in response to repeated apheresis. Using published data from patients with elevated Lp(a) and otherwise normal lipid metabolism, average FCR’ for LDL-cholesterol was calculated to 0.1692 (95% CI 0.01676 to 0.3384) [day^− 1^], while FCR for healthy volunteers was calculated from [32] to 0.35 (95% CI 0.10 to 0.68) [day^− 1^]. Respective SR’ were calculated from FCR’ according to Eq. 3 (Table 2).

**Table 3:** Turnover data calculated after first H.E.L.P. apheresis. As occurring or worsening edema during therapy interferes with calculating the plasma volume, only data from each patient’s first apheresis were considered (*n* = 6) for the calculation of apparent fractional catabolic rates (FCR’) and apparent synthetic rate (SR’). Each individual FCR’ and SR’ are given as mean calculated from all available blood samples after first apheresis, calculations were done according to Eqs. 2 and 3 (see methods for details)

Patient	Serum apoB		Serum cholesterol		LDL-cholesterol		Plasmavolume [1]	Body weight [kg]
	FCR’ (day^−1^)	SR’ (mg*kg^− 1^*day^− 1^)	FCR’ (day^− 1^)	SR’ (mg*kg^− 1^*day^− 1^)	FCR’ (day^− 1^)	SR’ (mg*kg^− 1^*day^− 1^)		
1	0.46	21.9	0.78	76.9	0.35	19.2	3.034	81
2	0.51	18.5	0.70	48.4	0.12	4.7	3.009	84
3	0.34	15.6	0.27	23.8	0.24	12.3	2.584	71
4	1.04	35.5	1.41	125.6	0.67	27.0	2.504	61
5	0.82	31.3	0.76	64.9	0.62	21.7	2.872	80
6	0.98	30.6	1.09	104.5	0.60	17.7	2.956	59
Mean ± SD	0.69 ± 0.29	25.6 ± 8.0	0.84 ± 0.39	74.0 ± 37.0	0.43 ± 0.23	17.1 ± 7.7		

### Lipoprotein profile before and after apheresis

**Fig. 4:**
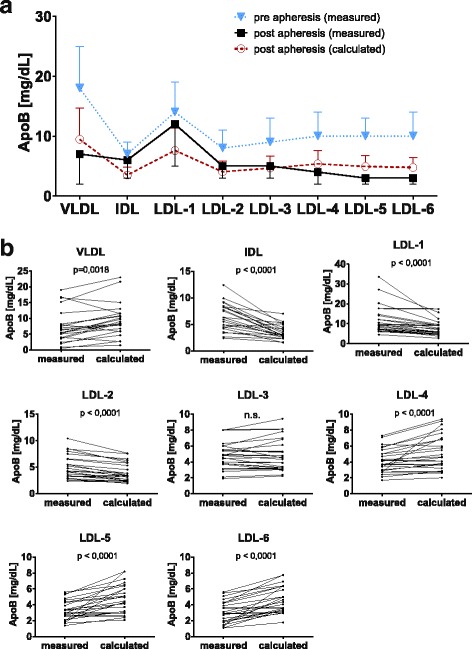
Lipoprotein profile before and after apheresis. Lipoproteins pre and post apheresis were separated into their density classes and LDL were divided into 6 subclasses by further ultracentrifugation. Density increases from left to right (see methods for details). **a**: Measured ApoB values (mean ± SD of 6 patients) are given as closed symbols (measured pre-apheresis values: closed triangles; measured post-apheresis levels: closed squares). Expected post apheresis values calculated by apheresis efficiency are given in open red circles. **b**: Comparison of measured and calculated post-apheresis levels by Wilcoxon matched pairs test, a value of *p* < 0.05 was considered statistically significant

As H.E.L.P.-apheresis eliminates virtually all ApoB-containing lipoproteins entering the plasma circuit [25], all ApoB-lipoproteins should be similarly reduced, regardless of their lipid class. We calculated the theoretical lipid profile after apheresis based on overall ApoB-elimination. Figure 4a shows the averaged lipoprotein profile before and after apheresis as well as expected calculated post-apheresis values. All lipoprotein fractions were reduced by apheresis, but the observed profiles significantly differed from the expected profiles according to calculations when analysed in a matched pairs test (Fig. 4b). Within the LDL subfractions, the profile shifted to larger LDL-subclasses. VLDL and small-dense LDL subclasses LDL-5 and LDL-6 were lower in comparison to the calculated post-apheresis values, whereas, IDL and especially LDL-1 were significantly higher than the expected post-apheresis values.

**Fig. 5:**
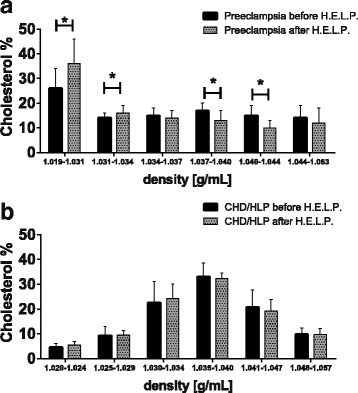
Cholesterol distribution in LDL-subfractions after H.E.L.P. apheresis in PE patients (**a**) compared with data derived from literature [33] (**b**). Cholesterol in the different subclasses is given in % as mean ± SD of total LDL-Cholesterol (set as 100%). *p < 0.05 with Holm-Sidak-Test. (Note, that the density ranges of individual subclasses are not identical in Fig. 5a and b)

The percentaged cholesterol distribution among different subclasses of LDL pre- and post-apheresis is shown in Fig. 5a for PE patients. As shown in Fig. 4 for ApoB, we observed a shift in the cholesterol distribution among LDL-subclasses to buoyant subclasses in our PE patients. In PE, proportions of the larger LDL subclasses LDL-1 and LDL-2 were significantly higher and proportions of denser LDL-subclasses LDL-4 and LDL-5 were significantly lower post-apheresis than pre-apheresis. As control data from healthy pregnant women undergoing apheresis therapy are not available, we compared our data to that from patients with coronary heart disease (CHD)/hyperlipidemia undergoing H.E.L.P.-apheresis (Fig. 5b) [33]. In accordance with theory, CHD/hyperlipidemia patients exhibited no apheresis-induced shift in lipid profiles; changes in the relative content of cholesterol were minimal and not statistically significant. Taken together, our PE patients demonstrated significantly increased lipid dynamics and apheresis altered the lipoprotein profile to a remnant removal disease like lipoprotein profile seen before in late onset PE [13].

## Discussion

In the “Freiburg Preeclampsia H.E.L.P. Apheresis study”, six women with early-onset PE (gestational week 24–27) were treated with H.E.L.P.- apheresis to ameliorate preeclamptic symptoms and prolong pregnancies [18]. That study was based on an earlier study by Wang et al. [17]. Other successful approaches to prolong pregnancies complicated by PE used DSC-apheresis [15, 16]. Both apheresis techniques were originally designed to lower LDL-cholesterol and significantly lower plasma lipid levels. While clinical aspects were discussed earlier [18], the present work focusses on in-depth data analyses of changes in lipoprotein profiles induced by apheresis and their implications for lipid metabolism in PE.

As is standard for lipid apheresis, ApoB and LDL-cholesterol were significantly reduced (Fig. 1), but to a substantially lower amount than reported in conjunction with H.E.L.P.-treatments for other pathologies and expected regarding treated plasma volume (Table 2). While rebound of ApoB and LDL-cholesterol levels take many days in other conditions [28, 34], rebound is much faster in our patients with PE (Fig. 3). Significant production of ApoB respectively VLDL during apheresis therapy (~ 2 h) may lead to the apparent reduced apheresis efficiency observed. In response to fetal demands, triglycerides and cholesterol increase in all lipoprotein fractions during normal pregnancy, and increased VLDL synthesis is likely during normal pregnancy. A major limitation of this study is the lack of appropriate control data from healthy pregnancies, but performing apheresis on healthy pregnant woman is out of question. A single case report describes one pregnant woman with heterozygous FH receiving LDL-apheresis (cascade filtration) during an otherwise normal pregnancy. Repeated apheresis treatments at intervals of 2 to 6 days decreased lipid levels gradually over the first three weeks down to 48% of LDL-cholesterol and 77% of serum-ApoB [35]. In contrast, we did not observe a sustainable decrease in cholesterol levels in our patients despite even shorter apheresis sequences. Another case report on a mother with FH and otherwise healthy pregnancy reported stable LDL-cholesterol levels by weekly apheresis, but ~ 2 plasma volumes were treated, leading to an overall elimination efficiency of ~ 80% [36]. Since FH is generally characterized by impaired LDL elimination but not by reduced VLDL production [37, 38], ApoB respective VLDL production and turnover to LDL are probably much higher in our patients. There is unfortunately no other data on the lipid levels of normotensive pregnant women treated by apheresis available, and it cannot be ruled out that the differences between these two described pregnancies and our patients with PE may be individual.

Due to their severe condition and advanced pregnancies, our patients were not necessarily in a fasting state when blood was sampled or apheresis was performed, introducing a putative bias. Total cholesterol, LDL cholesterol and Apo B remain stable during standard meal as well as in the experimental setting of an extreme nutritional fat load [39]. Not surprisingly, significant changes are seen only in total triglyceride, peeking ~ 4 h after food intake and going down after 6 h. Interestingly, with standard meal, maximal triglyceride level did not exceed 150% of baseline level, and even under extreme fat load, maximal triglyceride level was < 200% of baseline. Given the severe clinical condition of our PE patients, nutritional intake of lipid rich food is supposed to be rather low. Thus the putative bias of the non-fasting state of our patients on triglyceride levels was considered low, which is reflected by similar apheresis efficiency for triglycerides, LDL cholesterol and Apo B (Table 2). Whether the used ApoB essay discriminates between Apo B100 and Apo B48 is not known, but since the amount of Apo B48 in the postprandial state is < 1% of Apo B100 [39], a possible bias can be neglected for Apo B concentrations. Calculations and discussion focused on total cholesterol, LDL cholesterol and ApoB levels where a bias due to non-fasting conditions can be neglected.

Although mean data revealed no significant difference between FCR and SR calculated from tracer kinetics (ApoB SR 13.7 ± 4.2 mg*kg^− 1^*day^− 1^) and rebound data (18.9 ± 6.0 mg*kg^− 1^*day^− 1^), FCR’ and SR’ data calculated from rebound after apheresis did not necessarily correlate with tracer kinetic data on the individual level [28]. Since tracer kinetic data are regarded as gold standard, individual SR and FCR calculated from rebound after apheresis should be interpreted with caution, and rebound derived FCR and SR were regarded as apparent FCR’ and SR’. Tracer kinetics revealed that ApoB SR and FCR are unaffected by apheresis, and SR for ApoB in patients with FH had been estimated to 13.9 ± 4.9 mg*kg^− 1^*day^− 1^ [40]. For our patients, apparent SR’ were 25.6 ± 8.0 mg*kg^− 1^*day^− 1^ for ApoB and 17.1 ± 7.7 mg*kg^− 1^*day^− 1^ for LDL-cholesterol, findings in line with earlier data calculated by Wang et al. [17]. If rebound data only are compared, rebound in our patients was much faster than that in healthy individuals (Fig. 3b), but all these studies suffer from small numbers and high individual variations. Nevertheless, lipoprotein turnover seems to be highly accelerated in PE patients. Whether this is more pronounced in PE than in normal pregnancies, or whether pregnancies having high lipoprotein turnover are more at risk for developing PE cannot be concluded from the available data so far, but access to a healthy pregnant control group receiving apheresis is utterly impossible. Nevertheless, the differences in lipoprotein profiles observed between PE patients and normotensive pregnant controls in late gestation [13] are well in line with the discussed imbalances described in this study.

Heparin is administered before and during apheresis. Heparin liberates lipoprotein lipase (LPL), hepatic lipase (HL) and endothelial lipase from the liver and other tissues [41], and the liberated lipases contribute to the conversion of lipoproteins. Higher LPL activity is associated with a shift to IDL and larger LDL [42]. HL deficiency is associated with the accumulation of buoyant LDL, whereas increased HL activity leads to increased small dense LDL (LDL-5 and LDL-6 in Fig. 4) [13, 43]. Exposure of VLDL to liberated LPL could explain the higher than expected level of IDL, but in analogy, liberated hepatic lipase should lead to enriched small-dense LDL, but enriched larger LDL-1 fractions are observed instead. In normal pregnancy, LPL and HL activities are reduced, leading to an increase in TG in lipoproteins [8]. An increase in VLDL synthesis in PE apparently overflows decreased HL activity, and TG rich lipoproteins accumulate. Concordantly, pregnancies with high TG levels are at higher risk for PE [12, 13].

Upon detailed analysis, the difference between measured and calculated ApoB in the VLDL fraction is less pronounced than that difference in IDL and LDL-1 fractions (Fig. 4). This may be interpreted as a rapid interconversion of the produced nascent VLDL into large buoyant LDL-1 via IDL, with a rate-limiting step beyond LDL-1 interconversion. Since the filtrate after the precipitation is practically ApoB-free [25, 44], there is no difference in the removal efficiency of different ApoB-containing lipoproteins through the apheresis procedure itself. In contrast to our patients, a decreased conversion of VLDL to LDL was reported for FH patients after apheresis [40], and those patients exhibited no distinct abnormalities in lipoprotein subclasses after apheresis. A slight shift in the LDL-subclasses has been described in patients with CHD/hyperlipidemia [33], but the apheresis-induced shift to large buoyant LDL is much more pronounced in our patients (Fig. 5), supporting the hypothesis of accelerated synthesis and fast interconversion of VLDL to LDL-1, but not further down the LDL cascade. In our early-onset PE patients, the observed lipoprotein profile after apheresis resembles a remnant disease like lipoprotein profile, already described in conjunction with late-onset PE [13] and diabetic women preceding PE’s onset [45]. Perhaps an underlying remnant removal disorder has not yet exerted its full effect on lipoprotein profiles and becomes more obvious during ongoing gestation in PE. Interestingly, chylomicron remnants and VLDL compete for common clearance pathways [46], thus overproduction of VLDL may lead to the accumulation of chylomicron remnants in the postprandial state. While nascent large chylomicrons cannot penetrate the vessel wall, small circulating chylomicron remnants become a substrate for transendothelial transport and are suspected to deliver high amounts of cholesterol into the subendothelial space, leading to endothelial dysfunction and CVD [47, 48]. Accumulation of cholesterol during PE in the vessel wall is well in line with the observation that Patients who suffered from PE have a higher risk of developing CVD in later life [12]. An underlying remnant removal disease like metabolic imbalance in PE may provide a physiological link between unmet fetal demands and maternal endothelial damage.

Imbalances of lipid metabolism have been linked to PE for many years, leading to the early notion that lipid apheresis may be beneficial in severe PE [13]. Lipid-apheresis generally reduces not only ApoB-containing lipoproteins, but also a wide variety of pro-inflammatory and rheologically-active substances. Some authors attributed the beneficial effects of apheresis to the slight elimination of sFlt rather than to the elimination of lipoproteins [16], but H.E.L.P.-apheresis does not reduce sFlt-levels [18, 23]. Regarding the fast lipoprotein rebound described herein, the benefits of lipid-apheresis in a multifactorial disease like PE are not likely to be attributable to a simple reduction in lipid levels alone; several other factors such as inflammatory mediators and rheology may play important roles as well.

## Conclusion

Detailed analyses of lipid profiles after apheresis support the hypothesis of a remnant disease like imbalanced lipid metabolism in PE [13]. VLDL synthesis seems to be highly accelerated, while the elimination and conversion to LDL beyond buoyant LDL-1 does not seem to match these dynamics. The possible accumulation of cholesterol-rich remnants during PE is in line with several other pathological observations. While pregnancy is a well-orchestrated metabolic symphony, PE is characterized by deadly dissonances. Lipid metabolism may be one of the musicians playing out of tune. More thorough assessments of lipid metabolism in PE are seriously warranted to identify sorely-needed treatment options.

## Abbreviations

ApoA1: Apolipoprotein A1
ApoB: Apolipoprotein B
CHD: Coronary heart disease
CI: Confidence interval
DSC: Dextrane-sulfate cellulose
FH: Familial hypercholesterolemia
H.E.L.P.: Heparin-mediated extracorporeal LDL-precipitation
HDL: High-density lipoprotein
HL: Hepatic lipase
IDL: Intermediate-density lipoprotein
LDL: Low-density lipoprotein
Lp(a): Apolipoprotein(a)
PE: Preeclampsia
PlGF: Placental growth factor
sFlt: Soluble fms-like tyrosine kinase
VLDL: Very-low-density lipoprotein
